# Japanese Encephalitis Virus Surveillance in U.S. Army Installations in the Republic of Korea from 2021 to 2023

**DOI:** 10.3390/pathogens13080705

**Published:** 2024-08-20

**Authors:** Paula Lado, Gary P. Crispell, Sung Tae Chong, Myong Sun Kim, Ashley N. Esparza, Eric Zielinski, Akira Iwami, Kelly P. Williams, John J. Eads, Kei Jimbo, Dana N. Mitzel, Lee W. Cohnstaedt, Joshua B. Richardson, Jeffrey R. Kugelman, Craig A. Stoops

**Affiliations:** 1 National Bio and Agro-Defense Facility, USDA Agricultural Research Service (ARS), P.O. Box 1807, Manhattan, KS 66505, USA; paula.lado_henaise@usda.gov (P.L.); dana.mitzel@usda.gov (D.N.M.); lee.cohnstaedt@usda.gov (L.W.C.); 2 Environmental Molecular Biology Laboratory, U.S. Army Public Health Command-Pacific, Camp Zama, Zama City 252-0027, Kanagawa, Japan; gary.p.crispell.civ@health.mil (G.P.C.); ashley.n.esparza2.ctr@health.mil (A.N.E.); eric.m.zielinski.ctr@health.mil (E.Z.); akira.iwami2.ln@health.mil (A.I.); kelly.p.williams@gmail.com (K.P.W.); 3 Public Health, Environmental Health Section, Defense Health Agency Brian Allgood Army Community Hospital, Camp Humphreys 96271, Republic of Korea; sungtae.chong.ln@health.mil (S.T.C.); myongsun.kim4.ln@health.mil (M.S.K.); 4 Entomology, U.S. Army Public Health Command-Pacific, Camp Zama, Zama City 252-0027, Kanagawa, Japan; john.j.eads3.mil@health.mil (J.J.E.); kei.jimbo.ln@health.mil (K.J.); 5 Center for Genome Science, USAMRIID, U.S. Army Medical Research Institute of Infectious Diseases, Fort Detrick, MD 21702, USA; joshua.b.richardson2.civ@health.mil (J.B.R.); jeffrey.r.kugelman.mil@health.mil (J.R.K.)

**Keywords:** Japanese encephalitis, JEV genotype, *Culex*, Japanese encephalitis epidemiology, Republic of Korea, *Cx. bitaeniorhynchus*, *Cx. tritaeniorhynchus*, *Cx. pipiens*

## Abstract

Japanese encephalitis is a disease caused by the Japanese encephalitis virus (JEV) and is a concern for U.S. military personnel stationed in the Republic of Korea (ROK). The recent literature reports a potential shift from GI to GV as the dominant genotype circulating in east Asia. In the ROK, GV has been reported in a few *Culex* spp., but not in the main JEV vector, *Cx*. *tritaeniorhynchus*. The goal of this surveillance was to shed light on the current knowledge of the epidemiology of JEV in the ROK by analyzing mosquito collection data from three consecutive years, 2021–2023, and molecularly detecting and genotyping JEV in all *Culex* spp. collected in several military locations across the ROK. In this study, we detected only JEV GI in *Cx*. *tritaeniorhynchus* in 2021 samples. In contrast, all 2022 and 2023 positive samples were GV and detected in *Cx*. *bitaeniorhynchus*, *Cx*. *orientalis*, and *Cx*. *pipiens*. Results support a shift in JEV genotype in the ROK and suggest that for GV, *Culex* spp. other than *Cx*. *tritaeniorhynchus* may be playing an important role.

## 1. Introduction

Japanese encephalitis (JE) is a disease caused by the Japanese encephalitis virus (JEV), a mosquito-borne RNA *Flavivirus* distributed throughout southeast and east Asia. Japanese encephalitis has a yearly incidence of approximately 30,000–50,000 human encephalitis cases [1,2], causing 10,000–15,000 deaths [3]. In addition to the fatalities, the estimated global impact from JEV in 2002 was 709,000 disability-adjusted life years, although the estimates should be taken with caution due to the highly dynamic transmission of JE [3,4]. JEV is mainly transmitted by rice-paddy-breeding mosquitoes belonging to the *Culex* genus through an enzootic cycle that includes avian reservoir hosts and porcine species as amplifying hosts [5]. Humans are considered “dead end hosts” since they are not capable of infecting naive mosquitoes due to their low viremia [5]. Most human cases are restricted to Asia and the western Pacific region; however, JEV genetic material has been detected in *Culex pipiens* mosquitoes and birds in Italy [6,7]. Moreover, JEV geographic expansion has recently been reported, as southeastern Australia experienced an outbreak in 2022 [8,9]. These records underscore the importance of surveillance and preparedness to avoid the establishment of this disease in new geographic regions [10,11].

Japanese encephalitis is considered a serious health threat to U.S. military personnel stationed in the Republic of Korea (ROK), and vaccination against JEV is a requirement for all active-duty personnel spending more than 30 days in the ROK [12,13]. The U.S. military routinely conducts mosquito surveillance in the ROK to understand the risk of mosquito-borne pathogens and guide mosquito control efforts as installations and training sites are located around areas where mosquito populations are abundant. The primary vector of JEV, *Culex tritaeniorhynchus* Giles, is routinely collected as part of this surveillance, as many sites are located close to the species preferred habitat, rice fields [10,12,14].

There are five recognized JEV genotypes, GI–GV. The recent literature reports a potential shift in the dominant genotype circulating across east Asia, from GI to GV, as GV has recently been reported in several areas and mosquito species [1,10,11,15,16]. This genotype was originally identified in 1952 in Malaysia and was not detected again until 2009, 57 years later, in China, where it was detected in *Cx. tritaeniorhynchus* [16].

In the ROK, as part of the U.S. Department of Defense Global Emerging Infections Surveillance (GEIS) program, GV was first reported in *Culex bitaeniorhynchus* mosquitoes collected from Daeseongdong in 2010, with subsequent detection in *Culex orientalis* and *Culex pipiens* collected in the Gangwon and Gyeonggi provinces [1,10,15,17]. Nevertheless, GV has yet to be reported in *Cx*. *tritaeniorhynchus*, the well-established primary vector for JEV in Korea [10,18], suggesting that the epidemiology of GV may be different than that known for GI and GIII, previous dominant genotypes. In addition to GV detection in *Cx. bitaeniorhynchus*, *Cx. pipiens*, and *Cx. orientalis* field collected mosquitoes, this genotype has recently been reported in a clinical case in the ROK [19]. In this context, we hypothesize that (1) GV is currently the dominant genotype in the ROK; (2) GV epidemiology differs from GI and GIII, and that (3) *Cx. tritaeniorhynchus* may not act as primary vector of JEV GV in the ROK. In turn, *Cx. bitaeniorhynchus* and/or *Cx. pipiens* play a role as vectors. The result of three years, 2021–2023, of JEV vector surveillance and JEV screening on U.S. military installations are presented to shed light on the current knowledge of the epidemiology of JEV in the ROK.

## 2. Methods

### 2.1. Collection Sites

**Figure 1:**
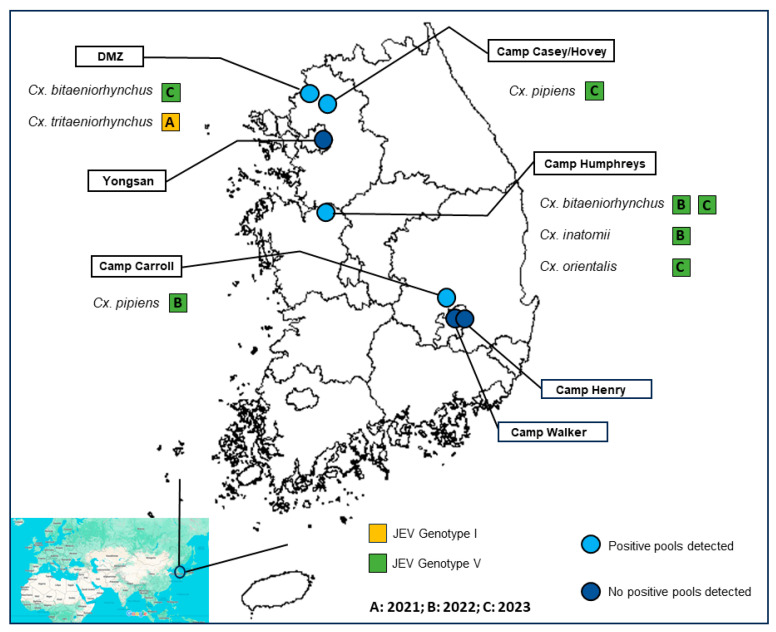
Map showing locations mosquito collection sites. Locations where positive pools where detected are shown in light blue circles, whereas locations where no positive pools were detected are shown in dark blue. For locations where positive pools were detected, the year and genotype are indicated by letters (A: 2021; B: 2022; C: 2023) and colors (yellow: JEV genotype I; green: JEV genotype V).

The main collection site sampled in this study was United States Army Garrison (USAG) Humphreys, also known as Camp Humphreys. (Figure 1). Camp Humphreys is located near the Anjeong-ri and Pyeongtaek metropolitan areas in the ROK. This area is characterized by wetland, rice fields, other agricultural farming, and the Anseong River, as described in [20]. At the center of the camp are small, isolated areas of unmanaged herbaceous vegetation, groves of trees, and a central drainage system, including water impoundments to reduce flooding [20]. A total of 12 traps at 12 different sites were placed within the camp (Table S1).

Mosquitoes were also collected at Daeseongdong inside the DMZ in 2021 and 2022 and from 2021 to 2023 along the DMZ on USAG Yongsan-Casey, including Camp Casey, Camp Hovey, Warrior Base, and Dagmar North Training Area. In the southern area of the ROK, mosquitoes were collected on USAG Daegu, including Camp Carroll, Camp Henry, and Camp Walker (Figure 1).

### 2.2. Mosquito Collection

At Camp Humphreys, adult mosquitoes were collected on a weekly basis from May to October during three consecutive years: 2021, 2022, and 2023. All mosquitoes in this geographic area were collected using mosquito magnet (MM) traps (Pro model, American Biophysics Corp., Greenwich, RI, USA), and traps were placed in the same specific locations across years. In 2021, weekly, and in 2022, monthly, collections using mosquito magnets were made in and around the DMZ at Daeseongdong village, Neutral Nations Support Camp (NNSC), the South Gate to the DMZ, Camp Bonifas, Warrior Base, and Dagmar North Training Area. Between May and September, from 2021 to 2023, mosquitoes were collected using a mix of CDC light traps (CDCLT), New Jersey (NJ) traps, BG-Sentinel (BG), and gravid traps (GT) on Camp Casey/Hovey, Yongsan, around DMZ at Dagmar North Training Area, Camp Henry, Camp Walker, and Camp Carroll. Collections were performed weekly or monthly, depending on the availability of resources.

After each collection event, the collected mosquitoes were removed from the traps and kept in a cooler while being transported to the lab for identification. Morphological identification of mosquitoes was performed using [21,22] keys. Once identified, mosquitoes were pooled by species (1–30 individuals per pool), transferred to 2 mL cryogenic vials, and kept in the freezer at −80 °C until further processing for JEV detection.

### 2.3. Molecular Detection of JEV and Genotyping

#### 2.3.1. Tissue Lysis and DNA/RNA Extraction

Nucleic acid isolation was performed using Zymo Direct-zol™-96 MagBead RNA kits (Zymo Research, Irvine, CA, USA) in conjunction with a Thermo Fisher Scientific KingFisher Flex Purification System (Thermo Fisher Scientific, Waltham, MA, USA). 400 uL of TRI reagent (Zymo) and (2) 3.2 mm stainless steel beads were added to each mosquito sample pool contained within a 1.5 mL microcentrifuge tube. Homogenization was performed utilizing a TissueLyser II (Qiagen, Germantown, MD, USA) set at 24 Hz/s for 7 min followed by centrifugation at 14,000× *g* for 10 min. 200 μL of the supernatant was then transferred to a 96-deep well plate, and 20 μL of Magbinding beads and 200 μL of 99.5% ethanol were added. Sample nucleic acids were eluted with 70 μL nuclease-free water. An extraction control of TRI reagent was used throughout the process. The KingFisher Flex instrument was programmed to follow the manufacturer’s recommendations for the Zymo Direct-zol™ kit (Zymo Research, Irvine, CA, USA). The eluant was temporarily stored at −20 °C until analysis.

#### 2.3.2. RT-PCR

Reverse-transcription polymerase chain reaction (RT-PCR) was performed using TaqMan Fast Virus Master Mix (Applied Biosystems, Foster City, CA, USA) following the manufacturer’s protocols, using 5 μL of mastermix per sample. The working dilutions of the forward and reverse primers were at a stock concentration of 10 μM and the probes at 5 μM. With the addition of a 5 μL sample, the total volume for each reaction was 20 μL. The ABI 7500 Fast Dx (Applied Biosystems) RT-PCR instrument was used and programmed with the following thermal cycling conditions: stage 1 (hold) 50 °C for 5 min, stage 2 (hold) 95 °C for 20 s, stage 3 (cycle) 95 °C for 3 s, and 60 °C for 30 s, repeated 40 cycles. Samples were initially screened using a JEV universal primer/probe set and then subsequently tested using primers and probes specific for GV (Table 1).

#### 2.3.3. Library Preparation Using TWIST Comprehensive Viral Research Panel

Comprehensive Viral Research Panel (Twist Biosciences, San Francisco, CA, USA) Library preparation was performed following the Twist Total Nucleic Acids Library Preparation EF Kit 2.0 for Viral Pathogen Detection and Characterization Workflow followed by the Twist Target Enrichment Workflow. Briefly, samples which had previously been determined positive via RT-PCR were diluted to 3.3 ng/uL. The samples were prepared using Random Primer 6 before cDNA synthesis (ProtoScript II First Strand cDNA Kit & NEBNext Ultra II Non-Directional RNA Second Strand Kit). cDNA was purified using 1.2xDNA purification beads, followed by fragmentation for 20 min at 37 °C with Frag/AT Enzyme + Buffer to obtain a 200–300 bp length. This kit incorporates DNA fragmentation, end repair, dA-tailing, and adapter ligation reagents and enzymes into a single reaction. Once fragmented, ligation of Twist Universal Adapters was performed by adding 2.5 uL of adapters and Ligation Master mix into the dA-tailed DNA fragments at 20 °C for 15 min. Samples were purified using DNA purification beads, and targeted segments were indexed using Twist UDI Primers. The indexed samples were purified, and the quality was assessed using the Tapestation 4150 and Qubit 4. The barcoded samples were combined into a single well to create a 500 ng pool. The library was then prepared for hybridization by using TWIST dry-down beads. The library was hybridized for 16 h to allow binding to streptavidin beads. Enrichment was performed via 23 cycles on the thermal cycler at 98 °C for 15 s, 60 °C for 30 s, and 72 °C for 30 s, and the product was purified using DNA purification beads and quality checked once again. The product was quantified using the NEBNext Library Quant Kit for Illumina (New England Biolabs, Ipswich, MA, USA) to dilute the library to a final concentration of 4 nM. The diluted library was loaded onto the MiSeq (Illumina) NGS platform using a 300-cycle kit. Sequencing reads were analyzed using the USAMRIID CGS in-house pathogen discovery pipeline, which assembles Illumina reads de-novo, then iteratively blasts contigs against the nt and nr databases. Reads were mapped to representative JEV genomes: GI (JF706279.1, strain M28), GIII (EF571853, strain Nakayama), and GV (MT568538.1, strain A18.3210) using minimap2 and its default short read settings [25].

## 3. Results

### 3.1. Mosquito Collection

#### 3.1.1. Camp Humphreys

Details of all *Culex* spp. mosquitoes collected are presented in Table 2. A total of 38,759 *Culex* spp. mosquitoes were collected at Camp Humphreys over the course of the study. The number of mosquitoes collected in 2022 and 2023 was similar (7896 and 6871, respectively) (Table 2). The *Culex* species recorded include *Cx. bitaeniorhynchus*, *Cx. inatomii*, *Cx. orientalis*, *Cx. pipiens,* and *Cx. tritaeniorhynchus*, and all five species were consistently found during the three years. The relative abundance, however, varied between years. In 2021, *Cx*. *bitaeniorhynchus* was the most abundant species (33.2%), whereas in 2022 and 2023 it was *Cx. inatomii* (52.2% and 47.8%, respectively). *Culex tritaeniorhynchus*, considered primary JEV vector in the ROK, represented 23.9% of the collections in 2021, 5% in 2022, and 17.3% in 2023 (Table 2).

#### 3.1.2. DMZ, Camp Casey/Hovey, Camp Henry, Camp Walker, Camp Carroll, and Yongsan

Details of all *Culex* spp. mosquitoes collected and corresponding trapping methods are presented in Table 2. A total of 58,265 *Culex* spp. mosquitoes were collected at non-Camp Humphreys locations over the course of the study (Table 3). The *Culex* species recorded include *Cx. bitaeniorhynchus* (14,932)*, Cx. inatomii* (1060), *Cx. orientalis* (8060)*, Cx. pipiens* (11,067), *Cx. tritaeniorhynchus* (23,099), and *Culex vagans* (47). All six species were consistently found across the three years, with the exception of *Cx. vagans. Culex vagans* abundance was generally low and was only found at the DMZ, Camp Casey/Hovey, and Yongsan locations in 2021 and 2022, with no collections in 2023 (Table 3).

### 3.2. Molecular Detection of JEV and Genotyping

A proportion of the collected *Culex* spp. mosquitoes were screened for JEV, and positive pools genotyped when genetic material was available.

#### 3.2.1. Camp Humphreys

At Camp Humphreys, a total of 860 *Culex* mosquito pools were tested, comprising 21,638 individual mosquitoes from the three collection years. No positive samples were detected for 2021 mosquito samples. In contrast, two pools tested positive for JEV from the 2022 samples and two pools from the 2023 collections. One of the 2022 positive pools was *Cx. bitaeniorhynchus* and the other *Cx. inatomii*. For 2023, one positive pool was *Cx. orientalis*, and the second one was *Cx. bitaeniorhynchus*. Genotyping revealed GV as the genotype present in all four pool samples (Table 1).

#### 3.2.2. DMZ, Camp Casey/Hovey, Camp Henry, Camp Walker, Camp Carroll, and Yongsan

Details of the mosquito species collected by year and location, together with JEV testing results for these locations, are presented in Table 2. Six *Culex* spp. were collected at these locations between 2021 and 2023: *Cx. bitaeniorhynchus*, *Cx. inatomii*, *Cx. orientalis*, *Cx. pipiens*, *Cx. tritaeniorhynchus*, and *Cx. vagans.*

In 2021, a total of 22,356 *Cx. tritaeniorhynchus* were collected at the DMZ. Of those, 8725 were tested in 344 pools, and 21/344 pools were positive for JEV (0.24 MIR) (Table 2). All 21 pools were genotyped as GI and corresponded to mosquitoes collected during September and October. Of the remaining mosquito species collected in 2021, all tested negative for JEV. In 2022, there was only one pool that tested positive for JEV. The pool was one of three *Cx. pipiens* pools tested from samples collected at Camp Carroll, and the genotype was identified as GV. In 2023, two pools tested positive for JEV: one pool from *Cx. bitaeniorhynchus* mosquitoes collected near the DMZ, and a second pool from *Cx. pipiens* mosquitoes collected at Camp Casey/Hovey. The genotype identified for both pools was GV.

It is worth noting that all positive pools from 2021 collections were mosquitoes collected using MM/NJ traps. In contrast, the 2022 positive *Cx. pipiens* pool included mosquitoes collected using CDCLT, and both 2023 positive pools correspond to females collected using GT.

## 4. Discussion

Surveillance plays a crucial role in vector-borne disease prevention and control. Considering changes in vector distributions and global travel, it is important to keep surveillance efforts active. Environmental changes (increase in temperature, rainfall) may lead to changes in the geographic distributional ranges of the main, currently recognized local vector species, *Cx. tritaeniorhynchus*, as well as in other species that are competent vectors of Japanese encephalitis. In the ROK and regionally, JEV genotype shifts and changes in dominance have been reported in recent years [10,18,26]. There is little information regarding the pathogenicity of JEV GV, and considerable discussion has been raised around the question of whether the JEV vaccine confers protection against GV and, if so, its effectiveness [10,27].

In light of the aforementioned information, there is a need to better understand the current epidemiology of JEV in the ROK, with the goal of informing and improving prevention and control strategies. In that context, this study analyzed mosquito surveillance data from three consecutive years in several locations across the ROK where U.S. military members, civilians, and family members work and live. These locations were also close to where 16 JE human cases were diagnosed between 2021 and 2023, according to the information available in the Korean Infectious Disease portal [28]. The surveillance was conducted as part of the Army’s Public Health program and the Defense Health Agency’s Global Emerging Infections Surveillance (GEIS) program to identify threats to Force Health Protection. As part of vector surveillance efforts, mosquitoes, including *Culex* spp., were collected using a variety of traps, tested for the presence of JEV, and then sequenced to identify the genotypes present. The traps have a collection bias, and this may have contributed to the differences in yearly collection abundance, species composition, and infection status of the collected mosquitoes. Similarly, different types of traps and trap numbers varied across years, which is one of the limitations of the study and prevents direct comparisons between years and collection sites.

A total of six *Culex* spp. were collected during this work: *Cx. bitaeniorhynchus*, *Cx. inatomii*, *Cx. orientalis*, *Cx. pipiens*, *Cx. tritaeniorhynchus*, and *Cx. vagans*. Of the 2021 mosquitoes collected and tested, all 21 positive pools were *Cx. tritaeniorhynchus* from the DMZ, and they were all identified as JEV GI. In that same year, despite a high number of *Cx. tritaeniorhynchus* collected on Camp Humphreys, all were negative for JEV. In 2022, on Camp Humphreys, JEV GV was detected in *Cx. bitaeniorhynchus* and *Cx. inatomii* collected using MM, and outside Humphreys, JEV GV was detected in *Cx. pipiens* on Camp Carroll collected using a CDCLT. JEV was also detected in four 2023 samples: one positive pool of each *Cx. orientalis* and *Cx. bitaeniorhynchus* collected in Camp Humphreys using MM, one positive pool of *Cx. bitaeniorhynchus* collected on Dagmar North Training areas near the DMZ using GT, and one positive pool of *Cx. pipiens* collected in Camp Casey/Hovey also using a GT. Thus, all 2021 positive pools were JEV GI, and positive pools of mosquitoes collected in 2022 and 2023 were identified as JEV GV, regardless of the mosquito species. These data support a shift in JEV genotypes in the ROK, which is consistent with the literature [10,18,26] and suggests that GV is currently the dominant genotype in this geographic area. Additionally, we report positive mosquito pools in females collected using GT. To our knowledge, this is the first report of JEV-positive samples captured utilizing GT in the ROK. This observation highlights the utility of these trapping devices and the potential for their use when, for example, manpower is limited and/or sorting, and identification time needs to be decreased. It is worth noting, however, that since these are blood-fed females, the virus could potentially be in the host’s blood rather than in mosquito tissues.

The presence of JEV was detected in several *Culex* spp.; including *Cx. bitaeniorhynchus*, *Cx. inatomii*, *Cx. orientalis*, *Cx. pipiens*, and *Cx. tritaeniorhynchus.* JEV has already been reported in most of these species which are considered competent vectors for JEV [29]. However, to the best of our knowledge, this is the first report of JEV in *Cx. inatomii*, and although its role as vector is unknown [30], it warrants further investigation. This finding also emphasizes the importance of surveillance. As JEV was detected in several *Culex* spp. across different geographic areas, it is possible that species other than the considered main local vector, *Cx. tritaeniorhynchus*, are playing a role in transmission and JEV epidemiology in the ROK. Recent reports of JEV GV in *Cx. bitaeniorhynchus* support this hypothesis, as suggested in [10]. It is possible that *Cx. tritaeniorhynchus* is the main vector for JEV GI (and likely GIII in the past), but that GV is transmitted preferentially by other species, such as *Cx. bitaeniorhynchus* and/or *Cx. pipiens*. Scenarios like this one could be relevant when we consider that *Culex* spp. have different distributions, and their abundance varies across regions.

Most disease models, rely on vector distribution data [14]. If GV is mainly being transmitted by *Cx. bitaeniorhynchus*, or by more than one species with different geographic distributions (for example, rice paddy vs. urban mosquitoes such as *Cx. pipiens*), then the models may not accurately reflect JEV epidemiology or risk areas in the ROK. For example, *Cx. pipiens* is distributed throughout the country whereas *Cx. tritaeniorhynchus* is found early in the mosquito season in the southern areas and moves northward as the season progresses and is greatly impacted by rainfall levels [31]. In 2015, the presence of JEV GV was reported by [1] in *Cx. orientalis* and *Cx. pipiens* for the first time in the ROK, although JEV GI had previously been reported in both species [5]. Those studies suggest that despite *Cx. pipiens pallens* displaying low competence in laboratory settings, they may play an active role in JEV epidemiology in Korea [1,5,32]. A study in Seoul showed that the population density of *Cx. tritaeniorhynchus* was less than 1% whereas that of *Cx. pipiens* was 60% and a different study showed that *Cx. pipiens* was over 90% of collections in urban areas [18,26]. This mosquito species has also been associated with pig farms [5]. Seasonality and species peak abundance are also different for various *Culex* spp., and this might be important since it is not clear whether *Cx. tritaeniorhynchus* abundance correlates to JE human cases. While [18] found no relationship between *Cx. tritaeniorhynchus* abundance and JE cases, [31] reports a correlation between JE cases and *Cx. tritaeniorhynchus* incidence. In [31], *Cx. pipiens* showed the highest prevalence during the first week of July, while *Cx. tritaeniorhynchus* peaked during the first week of September [31]. While no relationship was found between JE cases and *Cx. tritaeniorhynchus* abundance, the distribution of wading birds and the incidence rate of JE cases are correlated well, especially in cities [18]. This supports the potential role of *Cx. pipiens* in JEV epidemiology in the ROK, since it is extremely abundant in urban settings, and tend to feed on birds. Furthermore, a recent study investigating JE seroprevalence in Artiodactyla species found significant associations between increased human JE cases and increased serfoprevalence of JEV in goat kids, lambs, fawns and elk calves [33]. Taking these reports and investigations together, it would be valuable to further investigate the potential role of both *Cx. pipiens* (*pipiens palens* and *pipiens molestus*) and *Cx. bitaeniorhynchus* as local vectors of JEV, especially for GV. As for *Cx. orientalis*, the potential to play a role in JEV epidemiology is considered limited, since it is not a frequent human biter [1], although more research is needed in this regard. It would also be valuable to investigate the seroprevalence in different animal species based on the studies mentioned above and across different regions of the country to better understand JEV GV epidemiology at the local level.

At present, the available data and literature support the hypothesis of a JEV genotype shift in the ROK, and GV appears to be the dominant genotype found in *Culex* spp. Molecular data has shown that contemporary GV sequences have considerably diverged since 1952. The minimum intraclade similarity is lower than that of GI-GIV [10]. In this context, and considering that the vaccine is based on GIII, it would be valuable to have more data on vaccine efficacy against GV. Concerns about vaccine effectiveness are not discussed here since they have been addressed elsewhere [10,27,31]. Nevertheless, it should be noted that the results presented here, together with the report of a GV human clinical JE case [19], highlight the need for continued research.

## 5. Conclusions

In conclusion, our results support the hypothesis that species other than *Cx. tritaeniorhynchus*, such as *Cx. bitaeniorhynchus* and/or *Cx. pipiens*, have a potentially relevant role in JE epidemiology in the ROK, especially for JEV GV. This study reinforces the importance of surveillance and suggests that different JEV genotypes might be preferentially transmitted by different local vectors. We present supporting evidence of a JEV genotype shift from GI to GV; thus, it is crucial to better understand GV transmission and epidemiology within the ROK to further develop effective prevention and control plans to protect civilians and military personnel from the disease.

## Figures and Tables

**Table 1:** Primer and probe sequence information for JEV GI/GIII/GV used in this study, taken from [23,24].

Target	Primer/Probe	Sequence
JEV	Forward	5′-GGCTCTTATCACGTTCTTCAAGTTT-3′
	Reverse	5′-ACTAGTAAGATGTTTCATTGCCACACTCT-3′
	Probe	5′-ATTAGCCCCGACCAAGGCGCTTT-3′
JEV GI/GIII	Forward	5′-GGTCTGCAACCCAAACAAGAA-3′
	Reverse	5′-GCCAGCATGAAGGGTATTGACAT-3′
	GI Probe	5′-TTGTGGGAGGTCTAGCCGAGTTGG-3′
	GIII Probe	5′-TCGTAGGTGGTTTGGCCGAGTTG-3′
JEV GV	Forward	5′-TGCGACAAACAAGCCGTGTA-3′
	Reverse	5′-TTGCACTGACACAGATCTTCTACTTCT-3′
	GV Probe	5′-CGTTGCACGAGGACCAGGCACTC-3′

**Table 2:** *Culex* spp. mosquitoes collected in Camp Humphreys using the mosquito magnet throughout the study. *Culex* species, year of collection, total number of mosquitoes collected, total number of mosquitoes tested for JEV, number of positive pools and JEV genotype are presented. MIR: minimum infection rate (number of positive pools/total mosquitoes tested × 100).

*Culex* spp.	Year	Total Collected	Total Tested	# of Pools Tested	# Positive Pools (MIR)	JEV Genotype
*Cx. bitaeniorhynchus*	2021	7954	2000	71	0	
	2022	1400	1400	54	1, (0.07)	GV
	2023	939	939	43	1, (0.11)	GV
*Cx. inatomii*	2021	4469	1156	47	0	
	2022	4118	3857	131	1, (0.03)	GV
	2023	3281	3281	124	0	
*Cx. orientalis*	2021	977	316	17	0	
	2022	181	181	20	0	
	2023	67	67	14	1, (1.49)	GV
*Cx. pipiens*	2021	4857	1735	66	0	
	2022	1800	1800	73	0	
	2023	1394	1394	60	0	
*Cx. tritaeniorhynchus*	2021	5735	1925	70	0	
	2022	397	397	21	0	
	2023	1190	1190	49	0	

**Table 3:** *Culex* spp. mosquitoes collected in the DMZ, Camp Casey/Hovey, Camp Henry, Camp Walker, Yongsan, and Camp Carroll throughout the study. *Culex* species, year of collection, total number of mosquitoes collected, total number of mosquitoes tested for JEV, number of positive pools and JEV genotype are presented. MIR: minimum infection rate (number of positive pools/total mosquitoes tested × 100).

Culex spp.	Location	Year	Trap Type	Tot Collected	Tot Tested	# Pools Tested	# Positive Pools, (MIR)	JEV Genotype
*Cx. bitaeniorhynchus*	DMZ	2021	MM/NJ	14,651	3102	165	0	
		2022	MM/NJ	109	109	6	0	
		2023	NJ/GT	166	155	6	1, (0.65)	GV
	Camp Casey/Hovey	2021	NJ/BG	0	0	0	0	
		2022	CDCLT/NJ/BG	0	0	0	0	
		2023	GT	1	1	1	0	
	Camp Henry	2022	CDCLT/NJ	0	0	0	0	
		2023	GT/MM	0	0	0	0	
	Camp Walker	2022	CDCLT/NJ	0	0	0	0	
		2023	GT/MM	0	0	0	0	
	Yongsan	2021	MM	5	5	4	0	
	Camp Carroll	2022	CDCLT	0	0	0	0	
		2023	GT	0	0	0	0	
*Cx. inatomii*	DMZ	2021	MM/NJ	1036	33	14	0	
		2022	MM/NJ	2	2	1	0	
		2023	NJ/GT	1	1	1	0	
	Camp Casey/Hovey	2021	NJ/BG	0	0	0	0	
		2022	CDCLT/NJ/BG	1	1	1	0	
		2023	GT	0	0	0	0	
	Camp Henry	2022	CDCLT/NJ	0	0	0	0	
		2023	GT/MM	1	1	1	0	
	Camp Walker	2022	CDCLT/NJ	14	14	2	0	
		2023	GT/MM	1	1	1	0	
	Yongsan	2021	MM	4	4	4	0	
	Camp Carroll	2022	CDCLT	0	0	0	0	
		2023	GT	0	0	0	0	
*Cx. orientalis*	DMZ	2021	MM/NJ	7957	58	38	0	
		2022	MM/NJ	15	15	5	0	
		2023	NJ/GT	42	42	4	0	
	Camp Casey/Hovey	2021	NJ/BG	2	2	2	0	
		2022	CDCLT/NJ/BG	21	21	9	0	
		2023	GT	0	0	0	0	
	Camp Henry	2022	CDCLT/NJ	0	0	0	0	
		2023	GT/MM	1	1	1	0	
	Camp Walker	2022	CDCLT/NJ	0	0	0	0	
		2023	GT/MM	6	6	3	0	
	Yongsan	2021	MM	0	0	0	0	
	Camp Carroll	2022	CDCLT	15	2	2	0	
		2023	GT	1	1	1	0	
*Cx. pipiens*	DMZ	2021	MM/NJ	4499	324	94	0	
		2022	MM/NJ	16	16	5	0	
		2023	NJ/GT	276	217	17	0	
	Camp Casey/Hovey	2021	NJ/BG	7	7	6	0	
		2022	CDCLT/NJ/BG	104	104	26	0	
		2023	GT	30	30	16	1, (3.33)	GV
	Camp Henry	2022	CDCLT/NJ	96	9	3	0	
		2023	GT/MM	1516	1516	67	0	
	Camp Walker	2022	CDCLT/NJ	885	804	30	0	
		2023	GT/MM	741	741	40	0	
	Yongsan	2021	MM	2762	2742	112	0	
	Camp Carroll	2022	CDCLT	55	55	3	1, (1.82)	GV
		2023	GT	80	80	4	0	
*Cx. tritaeniorhynchus*	DMZ	2021	MM/NJ	22,356	8725	344	21, (0.24)	GI
		2022	MM/NJ	197	197	9	0	
		2023	NJ/GT	3	3	2	0	
	Camp Casey/Hovey	2021	NJ/BG	0	0	0	0	
		2022	CDCLT/NJ/BG	0	0	0	0	
		2023	GT	0	0	0	0	
	Camp Henry	2022	CDCLT/NJ	61	0	0	0	
		2023	GT/MM	189	189	13	0	
	Camp Walker	2022	CDCLT/NJ	20	1	1	0	
		2023	GT/MM	237	237	14	0	
	Yongsan	2021	MM	32	32	9	0	
	Camp Carroll	2022	CDCLT	4	4	1	0	
		2023	GT	0	0	0	0	
*Cx. vagans*	DMZ	2021	MM/NJ	8	8	4	0	
		2022	MM/NJ	3	1	1	0	
		2023	NJ/GT	0	0	0	0	
	Camp Casey/Hovey	2021	NJ/BG	0	0	0	0	
		2022	CDCLT/NJ/BG	21	21	7	0	
		2023	GT	0	0	0	0	
	Camp Henry	2022	CDCLT/NJ	0	0	0	0	
		2023	GT/MM	0	0	0	0	
	Camp Walker	2022	CDCLT/NJ	0	0	0	0	
		2023	GT/MM	0	0	0	0	
	Yongsan	2021	MM	15	15	1	0	
	Camp Carroll	2022	CDCLT	0	0	0	0	
		2023	GT	0	0	0	0	

## Data Availability

Sequences are available upon request.

## Supplementary Materials

The following supporting information can be downloaded at: https://www.mdpi.com/article/10.3390/pathogens13080705/s1, Table S1: Sampling sites at Camp Humphreys.
